# Retracted: Biomedical Implications of Heavy Metals Induced Imbalances in Redox Systems

**DOI:** 10.1155/2020/1913853

**Published:** 2020-11-27

**Authors:** BioMed Research International

BioMed Research International has retracted the article titled “Biomedical Implications of Heavy Metals Induced Imbalances in Redox Systems” [1]. The article was found to contain a substantial amount of material, without citation, from previously published articles, including the following sources:

- Danyal Ibrahim, Blake Froberg, Andrea Wolf, Daniel E. Rusyniak. "Heavy Metal Poisoning: Clinical Presentations and Pathophysiology", Clinics in Laboratory Medicine, 2006. 10.1016/j.cll.2006.02.003. [2]
- Robert A. Goyer and Thomas W. Clarkson, “Toxic Effects of Metals,” in casarett & doull's toxicology the basic science of poisons, 6th ed, Curtis D. Klaassen. https://www.biologicaldiversity.org/campaigns/get_the_lead_out/pdfs/health/Goyer_1996.pdf. [3]
- "Systems Biology of Free Radicals and Antioxidants", Ismail Laher, Springer Nature, 2014. 10.1007/978-3-642-30018-9. [4]
- Wikipedia contributors, "Mercury poisoning," Wikipedia, The Free Encyclopedia, https://en.wikipedia.org/w/index.php?title=Mercury_poisoning&oldid=919871578 (accessed April 11, 2019). [5]
- Jörg B. Schulz Allen I. Arieff, “Metabolic and Toxic Encephalopathies” in Neurological Disorders 2nd ed, Thomas Brandt, Louis R. Caplan, Johannes Dichgans, Christoph Diener Christopher Kennard, 2003. 10.1016/B978-012125831-3/50267-7. [6]
